# A Stochastic Version of the Brass PF Ratio Adjustment of Age-Specific Fertility Schedules

**DOI:** 10.1371/journal.pone.0023222

**Published:** 2011-08-04

**Authors:** Jack Baker, Adélamar Alcantara, Xiaomin Ruan

**Affiliations:** Geospatial and Population Studies, University of New Mexico, Albuquerque, New Mexico, United States of America; Universita' del Piemonte Orientale, Italy

## Abstract

Estimates of age-specific fertility rates based on survey data are known to suffer down-bias associated with incomplete reporting. Previously, William Brass (1964, 1965, 1968) proposed a series of adjustments of such data to reflect more appropriate levels of fertility through comparison with data on children-ever-born by age, a measure of cohort-specific cumulative fertility. His now widely-used Parity/Fertility or PF ratio method makes a number of strong assumptions, which have been the focus of an extended discussion in the literature on indirect estimation. However, while it is clear that the measures used in making adjusted age-specific fertility estimates with this method are captured with statistical uncertainty, little discussion of the nature of this uncertainty around PF-ratio based estimates of fertility has been entertained in the literature. Since both age-specific risk of childbearing and cumulative parity (children ever born) are measured with statistical uncertainty, an unknown credibility interval must surround every PF ratio-based estimate. Using the standard approach, this is unknown, limiting the ability to make statistical comparisons of fertility between groups or to understand stochasticity in population dynamics. This paper makes use of approaches applied to similar problems in engineering, the natural sciences, and decision analysis—often discussed under the title of uncertainty analysis or stochastic modeling—to characterize this uncertainty and to present a new method for making PF ratio-based fertility estimates with 95 percent uncertainty intervals. The implications for demographic analysis, between-group comparisons of fertility, and the field of statistical demography are explored.

## Introduction

Population dynamics are driven by the often complex interplay of demographic components of change including births, deaths, immigration, and emigration [1], [2], [3]. Obviously, modeling population change for purposes of either basic research or applied demography (population estimation or projection) requires adequate measures of these components [4], [5], [6]; however, their estimation can be highly challenging in developing settings where administrative infrastructures for capturing these data may be lacking [7], [8], [10]. Given the importance of such models for both public policy and basic scientific research on population, a strong motivation exists to either improve administrative data capture or develop methods for modeling population change with incomplete data [1], [8] In spite of its shortcomings, development of models is much less problematic than reforming administrative data capture and this reality has led to the development of numerous methods for modeling population change with incomplete or missing data [8], [10], [11], [12]. Given its importance to both public policy as well as short-term changes in population growth rates and age-structure [1], [12], [13] methods for estimating age-specific and total fertility rates (see glossary—Figure S1—for terms used in this paper) using incomplete and clearly under-reported survey data have enjoyed particular prominence in the literature on indirect estimation [1], [4], [8], [11], [14], [15], [16], [17].

**Figure 1:**
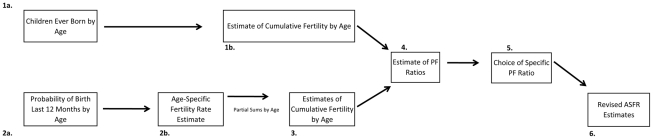
The Brass Parity/Fertility ratio method uses data on the risk of births in the 12 months prior (2a in the Figure) and children-ever-born by age (1a) to arrive at adjusted fertility estimates in light of underreporting in survey data.

As early as 1964, William Brass suggested the possibility that given an assumption that underreporting is equivalent across age groups, period measures of age-specific fertility could be adjusted by leveraging information on cohort parity such as the average number of children ever born to a woman of a given age [7]. Since that time, Brass and others have developed a number of methods for accomplishing such adjustments [10], [15], [16]. The proposed procedure is straightforward and summarized in Figure 1. First, alternative estimates of parity (cumulative fertility) by age are made from two data sources: (1) data on the average number of children ever born by age and (2) partial sums of survey-reported age-specific fertility rates (made using reported births from the previous year). These are represented in steps 1a. and 2a.–2b. in Figure 1. Age-specific fertility rates are summed up to each age-group of interest to estimate cumulative fertility up to each age group (step 3), while data on children ever born represent direct estimates of the cumulative fertility of cohorts up to that age (step 1b). The first estimate represents a period measure of fertility, while the latter estimate is a measure of cohort-specific fertility experience of the population and a direct measure of the expected *level* of fertility achieved by a specific age [8], [14], [15], [16], [18]. If fertility reporting is complete in survey data and no temporal trend (cohort effect) in fertility is observed [10] then the two measures should be approximately equal and sum to an equivalent total fertility rate. Where not, underreporting is suggested when the observed cumulative fertility in the period data is less than the average cohort parity in the age group of interest. Armed with these two sources of information on cumulative fertility, parity/fertility ratios (children ever born/survey-based estimate of parity by age) are computed for each age-group (step 4 in Figure 1), one of which is chosen to adjust observed estimates of age-specific fertility (Step 5). The adjusted estimates are then made (Step 6), which have an identical age-specific patterning of fertility (from the period data) but a higher overall *level* of achieved fertility captured in the cohort measures [1], [8], [14]. The method is presumed to remediate the down-bias in the magnitude of fertility measures obtained in survey data, given the assumption that children ever born data are more reliably recalled in general [8].

While there is no set algorithm for determining which PF ratio to use [12], [18], most demographers have suggested that using the PF ratio for younger age groups might tend to produce unreliable estimates since a greater proportion of women will not have experienced childbirth [1], [8], [18]. Likewise, since parity measures include the cumulation of complete fertility histories, it has also been suggested that use of later ratios might be biased by incomplete recall of birth histories among older women [8]. The United Nations' Manual X [8] suggests the use of P2/F2 (20–24), but a general guideline of choosing the P3/F3 (25–29) ratio as a viable alternative has also been repeatedly entertained [1], [8], [9], [14]. In practice, demographers often depend on review of the data to choose a reasonable alternative when the ratio of adjustments shows an age-specific patterning [10], [18]. In spite of the lack of a clear decision criteria, demographic intuition is often thought to provide a reliable indicator upon which to choose an appropriate PF ratio [1], [18].

**Figure 2:**
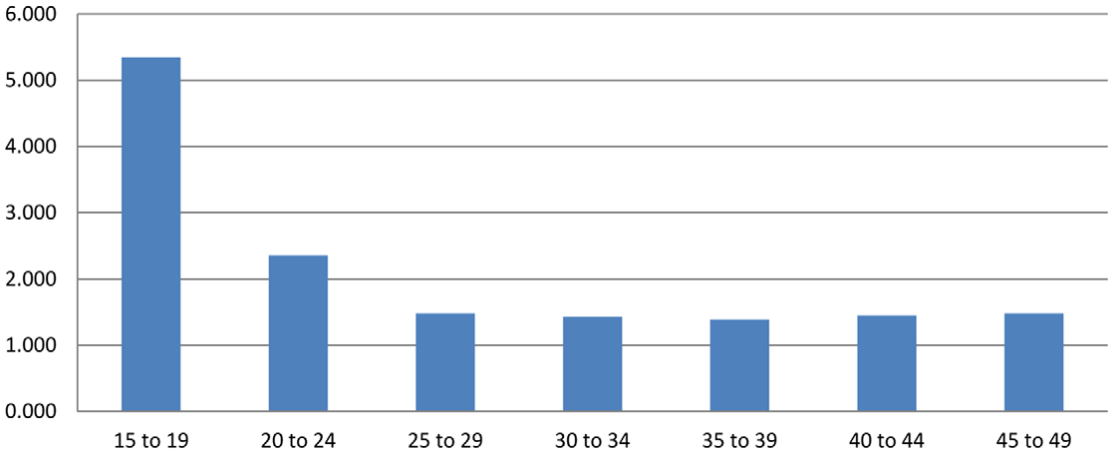
The Brass PF ratios are larger in earlier age intervals, but tend to stabilize in later age-intervals. Previous demographers have suggested the use of later ratios in light of such trends.

**Figure 3:**
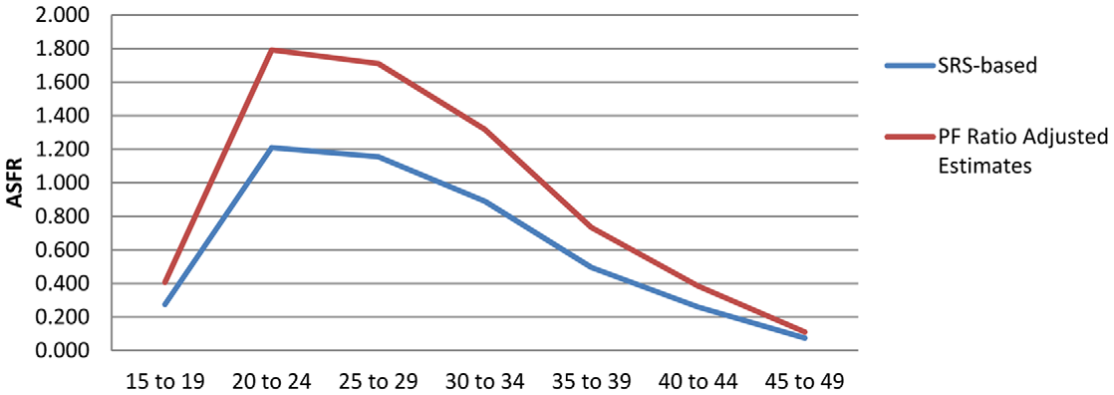
The Brass PF ratio adjustment scales up achieved fertility at each age level while preserving the age-specific patterning of fertility.

Table 1 provides an example of this procedure for the Bihar Province of India, using a combination of data on age-specific fertility risk from the Sample Registration System of India (1996–1998 vintage) in conjunction with data on children-ever-born by age from the Demographic and Health Survey conducted in the province in 1998. The method corresponds to variant B3, reported in the UN Manual X [8], which involves use of survey data from two separate sources: one estimating age-specific fertility and the other estimating children-ever-born (see p. 30). Column 1 reports the SRS-based estimate of age-specific fertility, which corresponds to step 2b. in Figure 1. Column 2 presents cumulative fertility by age—an estimate of parity that corresponds to step 3 in Figure 1. Column 3 presents the DHS-based estimate of children-ever-born by age (Step 1b in Figure 1). Column 4 provides the PF ratio estimates for each five-year age interval from 15–19 to 45–49 [8], which are simply the ratios of column 2 values to column 3 values. These correspond to step 4 in Figure 1. Column 5 presents each ASFR from column 2, multiplied against the P3/F3 ratio (for ages 25–29) in column 4 to arrive at the PF-ratio adjusted estimate (step 6 in Figure 1) of age-specific fertility in Bihar (1996–1998). Figure 2 presents the PF ratios by age, which suggest that the P3/F3 ratio is reasonable and stable across the remaining age-intervals. These ratios suggest likely underreporting in the SRS-based fertility estimates, especially in younger age-intervals. The adjusted total fertility rate measure is very close to the children ever born measure reported in column 4 for women 45–49, reflecting the mechanics of the PF ratio procedure and suggesting its plausibility. While the overall age-specific patterning of fertility is retained in the adjusted estimates, the overall estimated level of childbearing between the two schedules is marked (Figure 3). A reasonable conclusion is that the PF ratio procedure improved estimates of age-specific fertility and total fertility rates, as argued in previous research [14], [15], [16], [10], [9], [1], [12], [19], [20].

**Table 1:** Example of Brass PF Ratio Method.

Bihar (1996–1998)					
Age	SRS ASFR	Estimated Cumulative Fertility	DHS Children Ever Born (Parity)	PF Ratio	Adjusted ASFR (P3/F3)
15 to 19	0.275	0.275	1.470	5.345	0.407
20 to 24	1.210	1.485	3.500	2.357	1.792
25 to 29	1.155	2.640	3.910	**1.481**	1.711
30 to 34	0.890	3.530	5.040	1.428	1.318
35 to 39	0.495	4.025	5.590	1.389	0.733
40 to 44	0.260	4.285	6.210	1.449	0.385
45 to 49	0.075	4.360	6.450	1.479	0.111
TFR	4.360	*		TFR	6.457

The simplicity and ease with which the Brass PF ratio method is implemented, its apparent ability to produce much more reasonable estimates of age-specific fertility rate and total fertility rate than unadjusted ones, and its promotion by the leading demographic research units involved in training demographers around the world [1], [8], [10] has led to its widespread use. The method, however, does depend upon a number of rather strong and important assumptions. The assumptions related to use of the PF Ratio method are discussed at length in the UN Manual X [8] and include the notions that mortality has little effect on the measurement of children ever born (no survivorship bias), that migration effects may be ignored, and that no temporal trend in fertility exists within the study population [10], [18] While the first effect may be minor [8] it is likely that ignoring migration effects may constitute a much stronger assumption, since in the short-term migrants may often display very different fertility patterns than long-time residents of a region. This effect, however, may decay over time [21], [22] as migrants adjust fertility to more closely match “native” conspecifics. An assumption of trivial migration effects is likely to be problematic in smaller populations, those experiencing significant recent migration, or those measured at the subnational level where large population sizes cannot drown out such effects [1], [8], [10], [21], [22]. The assumption of constant fertility is also strong in light of the ongoing demographic transition and it is worth noting that variants and reconceptualizations of the PF ratio procedure have been proposed that do not impose an assumption of constant fertility [10]. While recognizing that these strong assumptions are important considerations for demographers applying the Brass PF ratio method, this paper focuses on a largely unexplored challenge associated with applications of the method: an evaluation of the previously unconsidered statistical uncertainty associated with adjusted age-specific fertility estimates in the traditional application of the Brass method, especially the B3 variant provided in the influential Manual X [8].

### Unacknowledged Statistical Uncertainty in the Brass PF Ratio Method and a Potential Solution

This issue of statistical uncertainty in estimation of fertility using the PF ratio method has received little attention in the demographic literature. Given the data sources used in its construction, however, it seems an unavoidable fact that such statistical uncertainty is associated with the ratio. Estimates of age-specific fertility risk and children ever born by age are both measured through survey data in the case of variant B3, and sampling variability is contained in both. If we think of both estimates as inputs to the PF ratio estimate, then the adjusted age-specific fertility schedules represent an output into which the uncertainty associated with each input is propagated. This uncertainty has not received an adequate amount of attention within the demographic literature, perhaps because demographers remain unfamiliar with potential solutions to the challenge and often make little use of statistical theory in their work [23]. Analogous problems, however, arise in engineering and applications of dynamic modeling and a number of approaches have been developed to deal with them under the label of *uncertainty analysis or stochastic modeling* [24], [25], [26], [27], [28], [29]. As is the case with the PF-ratio adjusted estimates of fertility, often the challenge revolves around an evaluation of how model *inputs* formulated with uncertainty affect uncertainty in an *output* of interest [30]. This input/output framework permits applications of monte carlo simulation in which the probability distributions associated with inputs can be directly related to the level and distribution of uncertainty associated with output estimates [31], [32], [33], [34], [35], [36], [37], [38]. This type of monte carlo-based analysis of uncertainty has been undertaken in the literature on animal demography [39], [40], [41], [42], [43], but to our knowledge has not been previously applied to human demographic studies. Here, monte-carlo resampling algorithms are employed to estimate the uncertainty associated with estimates of age-specific fertility rates made using PF-ratio (variant 3) adjustments and to compute uncertainty intervals about the estimates.

**Figure 4:**
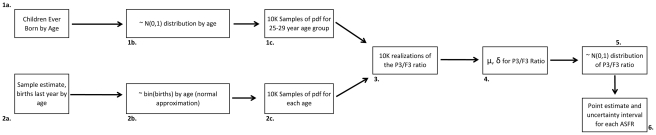
A stochastic version of the Brass PF ratio includes monte carlo simulation of the reported distributions of age-specific fertility risk and age-specific parity. This simulation allows consideration of statistical uncertainty and the construction of 95% upper and lower bounds of uncertainty around PF-ratio adjusted estimates of age-specific fertility.

The conceptual application of uncertainty analysis to the estimation of PF ratios with uncertainty intervals is illustrated in Figure 4. In the stochastic case, data on births by age for the previous year are used to estimate the binomial distribution of birth risk within each age interval from 15–19 to 45–49, as in step 2b. Here, the normal approximation is used. Likewise, the normal distribution of children-ever-born is estimated using survey data in step 1b. These steps contrast to those presented in the deterministic case illustrated in Figure 1 and Table 1, in which a single estimate is made for use in adjustments. Step 4 involves the computation of the mean and variance of the probability density function for the P3/F3 ratio to be used to adjust the survey-based estimates of age-specific fertility. This estimate is simulation based, conditioned on the inputs of two independent monte-carlo simulations involving resampling of 10,000 resamples of the estimated pdf of each input estimate (steps 1c and 2c): the survey-based estimate of age-specific fertility and the survey-based estimates of children-ever-born by age for the 25–29 year age group. This algorithm directly incorporates the uncertainty associated with each measure into the estimated statistical distribution associated with the P3/F3 ratio, represented in step 4 in Figure 4. The result in step 5 is a simulated estimate of the normal distribution of the P3/F3 ratio which is utilized in step 6 to estimate the point estimate of each age-specific fertility rate, *as well as an estimate of the 95% upper and lower confidence bounds for the estimate*. The end result of the procedure is a stochastic estimate of age-specific fertility that both adjusts for under-reporting while incorporating statistical information on uncertainty into the estimates. It provides a statistical distribution of ASFR to assess quality as well as to incorporate into stochastic projection models if desired [13], [39], [40], [44].

The purpose of this paper is to illustrate this novel approach for producing stochastic estimates of ASFR within the Brass PF ratio framework. The paper utilizes data from 5 randomly-selected Indian provinces and models age-specific fertility using data on children-ever-born from the Demographic and Health Surveys (www.measuredhs.com) and survey-based estimates of age-specific fertility from the Sample Registration System (SRS) of India (www.census.in). These data are utilized to illustrate the stochastic approach to the Brass method and provide point estimates and 95% upper and lower uncertainty bounds on these estimates using the exact process described in Figure 4. The results suggest the utility of further applications of this method and the implications of these findings for the practice of demography are reviewed.

## Materials and Methods

**Figure 5:**
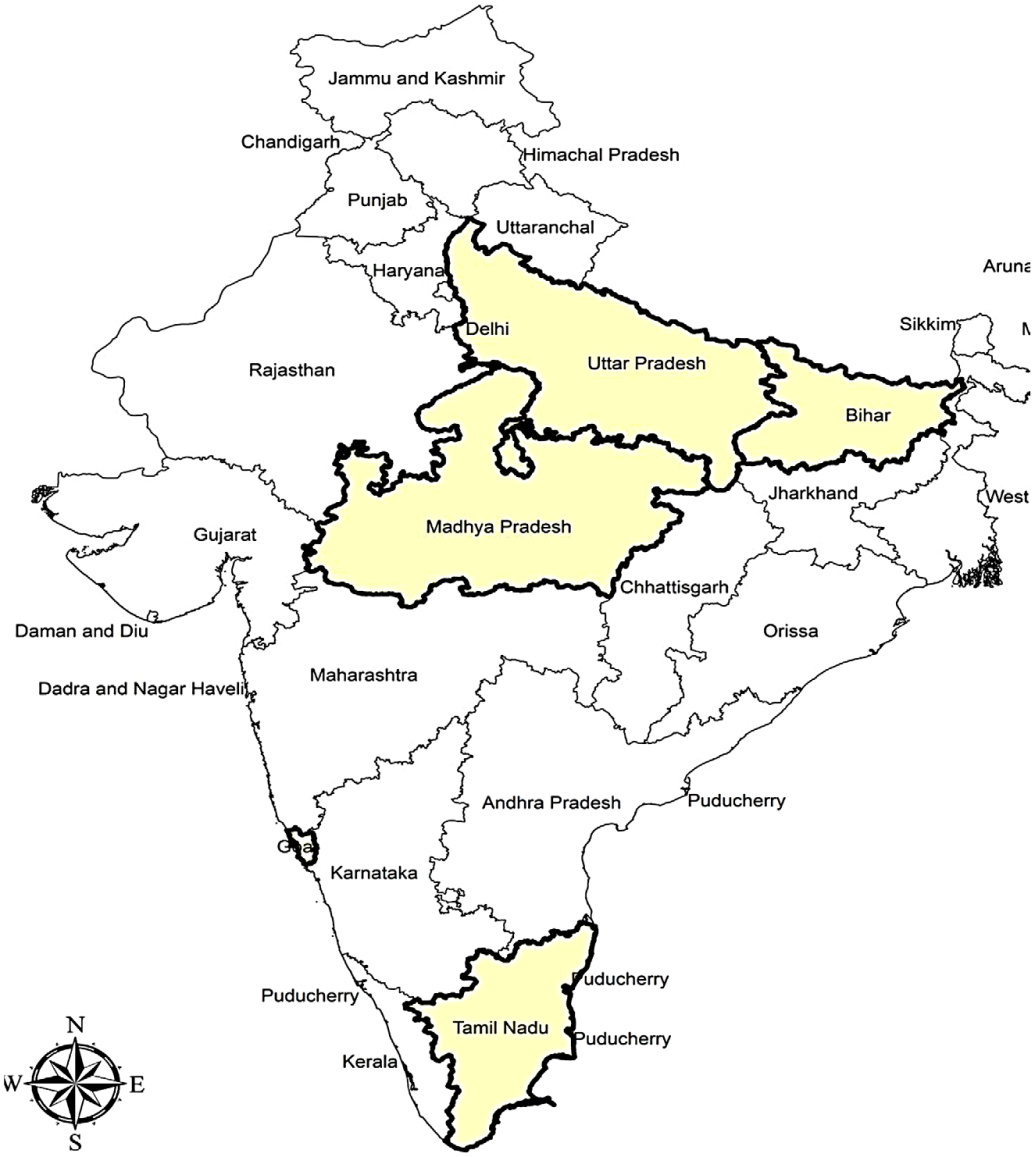
Five randomly-selected Indian provinces constitute the study area for this research.

A random sample of five Indian provinces was taken to include Bihar, Uttar Pradesh, Tamil Nadu, Madhya Pradesh, and Goa (Figure 5). Reported age-specific fertility estimates for 1997 were obtained from a series of reports made available by the Sample Registration System of India at (http://censusindia.gov.in). Data on children ever-born was obtained for each province using microdata from the associated Demographic and Health Surveys (www.measuredhs.com) for 1998. The one year gap between these datasets was considered trivial and unlikely to be of sufficient temporal duration to introduce bias into these estimates. Average children ever born by age were computed from the DHS data by the authors, including estimation of variance associated with each these estimates. The uncertainty associated with the SRS estimates is not publicly-reported, presenting a challenge for evaluating the uncertainty in PF ratio estimates associated with these data. As a reasonable substitute, we estimated variances associated with the binomial proportion of births in the last twelve months captured in the Demographic and Health Surveys, using the normal approximation [45]. Such surrogate evaluation of statistical properties of distributions has been previously utilized in a large number of uncertainty analyses [29], [39], [40], [41], [42], [46] and the binomial distribution has been argued to suitably reflect risk of birth in a number of previous studies [13], [43], [47], [48].

Using these data, PF ratios were computed, first using the standard procedure (Figure 1), then the stochastic algorithm (Figure 4). The trends of PF ratio by age were assessed graphically, using bar-charts (as in Figure 2). In all cases, significant graphical evidence of an age-trend in underreporting of fertility was observed, suggesting use of a later PF ratio in the adjustment. In this paper, the P3/F3 ratio was utilized in all analyses for consistency, in accordance with the recommendation of a number of previous published analyses [17], [18], [19] and its plausibility in light of graphical observations [1], [10], [18]. The associated adjusted age-specific fertility rate estimates, without uncertainty, were graphed and analyzed visually. Monte carlo simulations based on the normal distribution were employed to model the statistical distributions of age-specific and cohort-specific measures of parity. The normal approximation was used in simulating the binomial distribution of age-specific fertility risk [45], [49]. Tests of normality (Kolmogorov-Smirnov) were employed for each province's age-specific children-ever-born distributions, none of which rejected the null hypothesis of a normal distribution [49], [50]. As summarized in Figure 4 (steps 1c and 2c), monte carlo resampling was employed, involving 10,000 draws from the estimated ASFR distribution for ages 15–19, 20–24, and 25–29, then summation of these rates as the period measure of cumulative fertility by 25–29 years, followed by monte carlo resampling from the distribution of children ever born in the 25–29 age group. The associated distribution of the P3/F3 ratio was estimated using these inputs, recalculated at each round of the simulation, to 10 K draws. The resulting PF ratios were then used to adjust the SRS ASFR estimates and characterize the distribution of age-specific fertility distributions in each age-group with a point estimate and 95 percent upper and lower bounds of the uncertainty interval. All simulations were accomplished through original code written in the R statistical package. An annotated sample of this code is provided in Figure S2.

## Results

**Figure 6:**
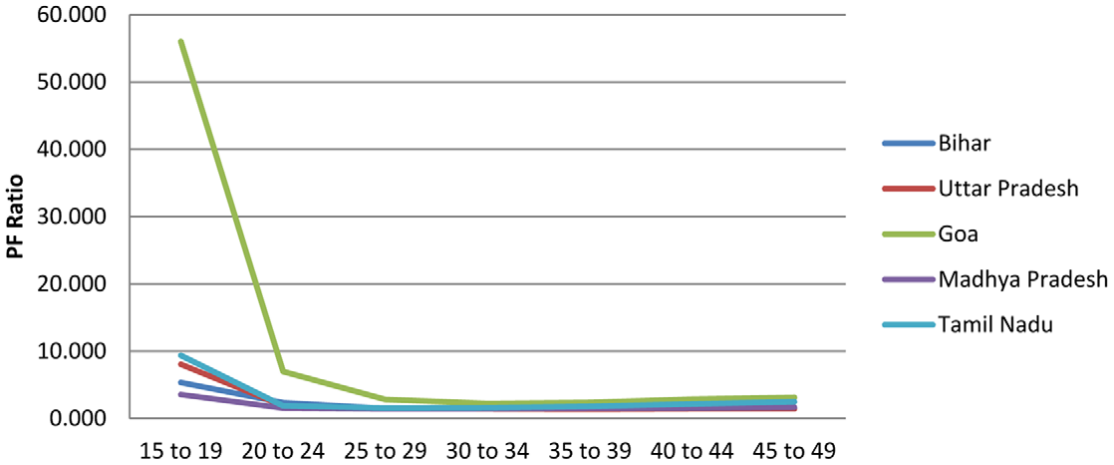
Parity/Fertility ratios vary across each of the Indian provinces included in this study; however, in all cases, they stabilize beyond the 25 to 29 year age interval.

The base results suggest that fertility underreporting is greater in younger age intervals and varies across provinces (Table 2). A consistent pattern across all provinces (Figure 6) is the rapid decline in suggested under-reporting from the 25 to 29 year age group forward, after which the PF ratios clearly stabilize. The level of incomplete reporting in the younger age intervals varies. In Goa, a very large PF ratio of 56 for the 15 to 19 year age group is likely related to small sample size in the DHS data with only 10 women interviewed. PF ratios for the youngest interval vary between 3.56 in Madhya Pradesh and 9.37 in Tamil Nadu. The clear convergence of PF ratios is apparent beyond the 20 to 24 age-group as in Figure 6 (see Tables 3, 4, 5, 6 also), which settle around reasonable levels at less than 2.0 [8], [18] for 4/5 provinces (the province of Goa was exceptional with a P3/F3 ratio of 2.834). The PF ratio-adjusted estimates lead to total fertility rates that are similar to the parity reported for the 45 to 49 year age group in the DHS question on children ever born in each case, reflecting both the mechanics of the method and the reasonability of the adjusted values in light of available data. Tables 3, 4, 5, 6 report results for all provinces except Bihar, which formed the example analysis and is reported in Table 1.

**Table 2:** Age-specific Parity/Fertility Ratios by Province.

Age	Bihar	Uttar Pradesh	Goa	Madha Pradesh	Tamil Nadu
15 to 19	5.345	8.055	56.000	3.557	9.374
20 to 24	2.357	1.894	6.981	1.556	1.882
25 to 29	1.481	1.442	2.834	1.416	1.557
30 to 34	1.428	1.415	2.213	1.430	1.587
35 to 39	1.389	1.379	2.378	1.505	1.808
40 to 44	1.449	1.435	2.873	1.547	2.162
45 to 49	1.479	1.432	3.137	1.659	2.485

**Table 3:** PF Ratio-Based Estimates of Age-Specific Fertility.

Uttar Pradesh (1996–1998)					
Age	SRS ASFR	Estimated Cumulative Fertility	DHS Children Ever Born (Parity)	PF Ratio	Adjusted ASFR (P3/F3)
15 to 19	0.200	0.200	1.611	8.055	0.288
20 to 24	1.240	1.440	2.728	1.894	1.788
25 to 29	1.355	2.795	4.030	**1.442**	1.954
30 to 34	0.990	3.785	5.355	1.415	1.427
35 to 39	0.575	4.360	6.012	1.379	0.829
40 to 44	0.310	4.670	6.702	1.435	0.447
45 to 49	0.115	4.785	6.854	1.432	0.166
TFR	4.785	*		TFR	6.899

**Table 4:** PF Ratio-Based Estimates of Age-Specific Fertility.

Goa (1996–1998)					
Age	SRS ASFR	Estimated Cumulative Fertility	DHS Children Ever Born (Parity)	PF Ratio	Adjusted ASFR (P3/F3)
15 to 19	0.025	0.025	1.400	56.000	0.071
20 to 24	0.295	0.320	2.234	6.981	0.836
25 to 29	0.550	0.870	2.466	**2.834**	1.559
30 to 34	0.370	1.240	2.744	2.213	1.049
35 to 39	0.140	1.380	3.282	2.378	0.397
40 to 44	0.035	1.415	4.066	2.873	0.099
45 to 49	0.005	1.420	4.455	3.137	0.014
TFR	1.420	*		TFR	4.025

**Table 5:** PF Ratio-Based Estimates of Age-Specific Fertility.

Madhya Pradesh (1996–1998)					
Age	SRS ASFR	Estimated Cumulative Fertility	DHS Children Ever Born (Parity)	PF Ratio	Adjusted ASFR (P3/F3)
15 to 19	0.440	0.440	1.565	3.557	0.623
20 to 24	1.375	1.815	2.825	1.556	1.947
25 to 29	1.070	2.885	4.086	**1.416**	1.515
30 to 34	0.630	3.515	5.025	1.430	0.892
35 to 39	0.315	3.830	5.763	1.505	0.446
40 to 44	0.140	3.970	6.141	1.547	0.198
45 to 49	0.025	3.995	6.626	1.659	0.035
TFR	3.995	*		TFR	5.658

**Table 6:** PF Ratio-Based Estimates of Age-Specific Fertility.

Tamil Nadu (1996–1998)					
Age	SRS ASFR	Estimated Cumulative Fertility	DHS Children Ever Born (Parity)	PF Ratio	Adjusted ASFR (P3/F3)
15 to 19	0.155	0.155	1.453	9.374	0.241
20 to 24	0.895	1.050	1.976	1.882	1.394
25 to 29	0.620	1.670	2.601	**1.557**	0.966
30 to 34	0.225	1.895	3.007	1.587	0.350
35 to 39	0.055	1.950	3.525	1.808	0.086
40 to 44	0.015	1.965	4.249	2.162	0.023
45 to 49	0.005	1.970	4.896	2.485	0.008
TFR	1.970	*		TFR	3.068

Tables 7, 8, 9, 10, 11 report the SRS-based ASFR estimates, the point estimates and standard deviations for the P3/F3 (25–29 years) ratios, and the adjusted ASFRs with 95 percent upper and lower bounds to these estimates based on the uncertainty analysis. While these adjusted estimates appear to appropriately remediate the suggested underreporting of fertility observed in the SRS data, they do not appear to artificially erase variation in observed fertility experience either across age-groups or between provinces. The standard PF ratio procedure produced an expected higher schedule of age-specific fertility than observed in the SRS (1997) data; however, the estimates based on uncertainty analysis led to *even* higher estimates of the overall level of fertility than were observed in the traditional application of the Brass method. Table 12 presents the observed differences in TFR for each province in the original PF ratio adjusted TFR estimates and those produced using the stochastic procedure supported here. These differences are pronounced; on average, the adjusted schedules using the original PF ratio procedure introduced a 1.915 child increase in TFR. The final adjusted TFRs made using the procedure implemented in this paper introduced an estimated average difference of 2.550 children. These are large differences suggesting either bias in the procedure as implemented here or even greater than anticipated incomplete reporting in the SRS data. In 3/5 cases the final adjusted uncertainty interval about the TFR estimate using the stochastic procedure did not encompass the age-specific children ever born measure from the DHS survey. In one case the interval did contain the DHS estimate and in one case (Tamil Nadu), the estimate was actually lower than observed in the DHS data.

**Table 7:** PF Ratio-Based Estimates of Age-Specific Fertility with Uncertainty Intervals.

Bihar (1996–1998)								
Age	SRS ASFR	Estimated Cumulative Fertility	DHS Children Ever Born (Parity)	P3/F3	s.d.	Adjusted ASFR	95 Percent Lower Bound	95 Percent Upper Bound
15 to 19	0.275	0.275	1.470	1.771	0.439	0.487	0.460	0.496
20 to 24	1.210	1.485	3.500			2.143	2.134	2.152
25 to 29	1.155	2.640	3.910			2.046	2.037	2.054
30 to 34	0.890	3.530	5.040			1.576	1.568	1.585
35 to 39	0.495	4.025	5.590			0.877	0.868	0.885
40 to 44	0.260	4.285	6.210			0.460	0.452	0.469
45 to 49	0.075	4.360	6.450			0.133	0.124	0.141
TFR	4.360	*			TFR	7.722	7.643	7.782

**Table 8:** PF Ratio-Based Estimates of Age-Specific Fertility with Uncertainty Intervals.

Uttar Pradesh (1996–1998)								
Age	SRS ASFR	Estimated Cumulative Fertility	DHS Children Ever Born (Parity)	P3/F3	s.d.	Adjusted ASFR	95 Percent Lower Bound	95 Percent Upper Bound
15 to 19	0.200	0.200	1.611	1.776	0.419	0.355	0.347	0.363
20 to 24	1.240	1.440	2.728			2.202	2.194	2.210
25 to 29	1.355	2.795	4.030			2.406	2.398	2.415
30 to 34	0.990	3.785	5.355			1.758	1.750	1.766
35 to 39	0.575	4.360	6.012			1.021	1.013	1.029
40 to 44	0.310	4.670	6.702			0.551	0.542	0.559
45 to 49	0.115	4.785	6.854			0.204	0.196	0.212
TFR	4.785	*			TFR	8.498	8.441	8.556

**Table 9:** PF Ratio-Based Estimates of Age-Specific Fertility with Uncertainty Intervals.

Goa (1996–1998)								
Age	SRS ASFR	Estimated Cumulative Fertility	DHS Children Ever Born (Parity)	P3/F3	s.d.	Adjusted ASFR	95 Percent Lower Bound	95 Percent Upper Bound
15 to 19	0.025	0.025	1.400	2.834	0.405	0.071	0.063	0.079
20 to 24	0.295	0.320	2.234			0.836	0.828	0.844
25 to 29	0.550	0.870	2.466			1.559	1.551	1.567
30 to 34	0.370	1.240	2.744			1.049	1.041	1.057
35 to 39	0.140	1.380	3.282			0.397	0.389	0.405
40 to 44	0.035	1.415	4.066			0.099	0.091	0.107
45 to 49	0.005	1.420	4.455			0.014	0.006	0.022
TFR	1.420	*			TFR	4.024	3.969	4.080

**Table 10:** PF Ratio-Based Estimates of Age-Specific Fertility with Uncertainty Intervals.

Mahya Pradesh (1996–1998)								
Age	SRS ASFR	Estimated Cumulative Fertility	DHS Children Ever Born (Parity)	P3/F3	s.d.	Adjusted ASFR	95 Percent Lower Bound	95 Percent Upper Bound
15 to 19	0.440	0.440	1.565	1.752	0.446	0.771	0.762	0.780
20 to 24	1.375	1.815	2.825			2.409	2.400	2.418
25 to 29	1.070	2.885	4.086			1.875	1.866	1.883
30 to 34	0.630	3.515	5.025			1.104	1.095	1.113
35 to 39	0.315	3.830	5.763			0.552	0.543	0.561
40 to 44	0.140	3.970	6.141			0.245	0.237	0.254
45 to 49	0.025	3.995	6.626			0.044	0.035	0.053
TFR	3.995	*			TFR	6.999	6.938	7.060

**Table 11:** PF Ratio-Based Estimates of Age-Specific Fertility with Uncertainty Intervals.

Tamil Nadu (1996–1998)								
Age	SRS ASFR	Estimated Cumulative Fertility	DHS Children Ever Born (Parity)	P3/F3	s.d.	Adjusted ASFR	95 Percent Lower Bound	95 Percent Upper Bound
15 to 19	0.155	0.155	1.453	2.287	0.796	0.354	0.339	0.370
20 to 24	0.895	1.050	1.976			2.047	2.031	2.062
25 to 29	0.620	1.670	2.601			1.418	1.402	1.434
30 to 34	0.225	1.895	3.007			0.515	0.499	0.530
35 to 39	0.055	1.950	3.525			0.126	0.110	0.141
40 to 44	0.015	1.965	4.249			0.034	0.019	0.050
45 to 49	0.005	1.970	4.896			0.011	−0.004	0.027
TFR	1.970	*			TFR	4.505	4.396	4.615

**Table 12:** Comparison of Total Fertility Rate Across Estimates.

Province	SRS TFR	Original Adjusted TFR	Stochastic PF Ratio Adjusted TFR	95% Lower Bound	95% Upper Bound	DHS Children Ever-Born 45–49
Bihar	4.360	6.457	7.722	7.661	7.782	6.45
Uttar Pradesh	4.785	6.899	8.498	8.441	8.556	6.85
Goa	1.420	4.025	4.024	3.969	4.080	4.46
Madhya Pradesh	3.995	5.658	6.999	6.938	7.060	6.63
Tamil Nadu	1.970	3.068	4.505	4.396	4.615	4.90

**Figure 7:**
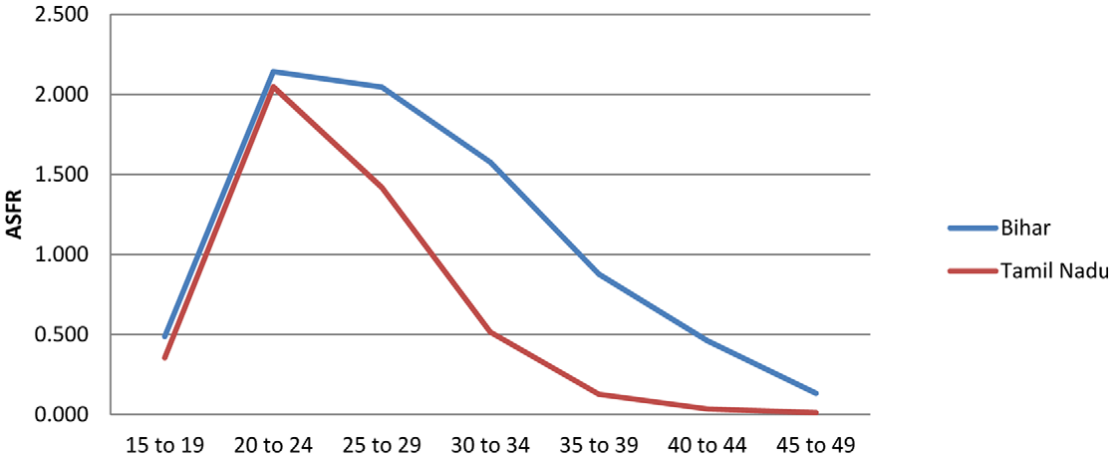
The stochastic procedure preserves diversity in age-specific fertility between the Provinces.

The 95 percent uncertainty intervals presented in Tables 7, 8, 9, 10, 11 are remarkably precise due to both the fairly large sample sizes associated with variance estimates using the DHS data and the large number of random samples drawn in the Monte Carlo simulation (see materials and methods section). However, this precision does not appear to have artificially erased the natural variation in fertility experience observed across the provinces. Figure 7 compares age-specific fertility curves for Bihar and Tamil Nadu. The estimates for Bihar still indicate much higher fertility than that observed in Tamil Nadu, just as in the original data where Bihar was reported with a TFR of 4.36 and Tamil Nadu with a TFR of 1.97. In the adjusted estimates, we observe TFRs of 7.69 and 4.51, respectively. These estimates vary in precision, in a way that appears natural as well. The range of estimates for Bihar is extremely tight, while in Tamil Nadu these ranges, and the shape of the ASFR curves in general, are more variable. These fluctuations in precision are a natural consequence of sample size differences rather than a systematic artifact of the monte carlo simulation.

## Discussion

The main contribution of this research has been to illustrate how the Brass PF ratio method may be extended to incorporate statistical uncertainty associated with data inputs using simple methods from stochastic modeling and uncertainty analysis. The results of the procedure provide point estimates and 95% uncertainty intervals for age-specific fertility that adjust survey-based estimates for underreporting of births. The approach represents a potentially significant improvement in stochastic demographic modeling, suitable for use in making between-group fertility comparisons as well as in demographic estimation or projection models for which a desire to incorporate statistical uncertainty exists. In the latter vein, there are natural links between this work and recent developments within the field of stochastic population forecasting [13], [44]. Curiously, most demographic modeling occurs without consideration of statistical uncertainty. In population forecasting, such uncertainty is often treated in an ad-hoc manner with low, high, and “most-likely” scenarios presented without uniform or robust description of what the proposed range of variation means in statistical terms [13]. In indirect estimation models, statistical uncertainty is more often than not simply ignored, as has historically been the case with the Brass PF ratio. It is curious that such little dialogue between statisticians and demographers has been undertaken [23] in spite of a clear common interest in the impact of missing data on the validity of estimates [8], [51] In statistical modeling, established methods for dealing with missing or incomplete data are standard fair [51], [52], as are indirect estimation methods such as the Brass PF ratio in demography [1], [8]. This research provides one potential example of an appropriate way to conduct such a dialogue between statisticians and demographers, bridging the two approaches within a common conceptual framework found within uncertainty analysis and stochastic modeling. This approach has a history in demographic studies of animal populations [12], [43], [47], [48] and clear potential for application to human demography as well.

**Figure 8:**
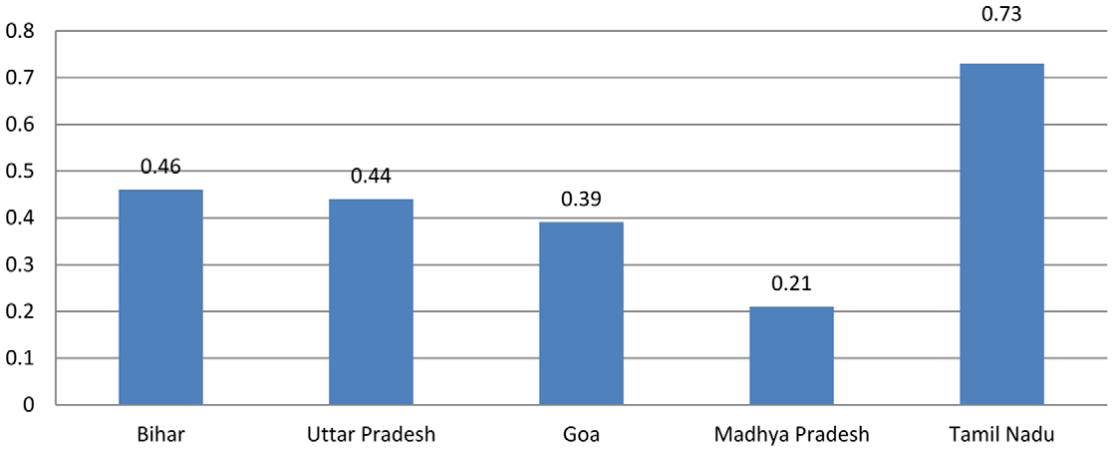
The stochastic procedure appears to make much higher estimates of age-specific and total fertility rates than observed in the standad Brass method. Thes have significant implications for population growth estimates.

From a practical point of view, it is clear that the results of the stochastic Brass PF ratio method differ in important ways from those found using the traditional procedure. First and foremost, the stochastic PF ratio-based estimates of age-specific fertility suggest much higher total fertility rates than those calculated using the deterministic procedure (Table 12). On average, the stochastic estimates suggest TFRs 1.128 children higher than the traditional Brass procedure and in all cases the 95% uncertainty interval associated with these estimates did not overlap the DHS-reported children-ever-born levels for women 45–49. These differences beg the question of whether the stochastic method overestimates fertility, or if the traditional Brass algorithm does not adjust it enough. Either eventuality is possible, but the higher TFRs implied by the stochastic procedure in comparison to the DHS data do raise the suspicion that this method might overstate fertility in general. To assess the sensitivity of population growth to these differences, we reviewed changes in asymptotic growth rates (Euler-Lotka R) [2], [3] associated with the observed range of fertility inputs while holding mortality levels constant at the National level and assuming net-migration of zero. This simple sensitivity analysis indicates the impact of these higher fertility estimates on models of population dynamics. Table 13 reports the results of this analysis, which suggests that the percent point difference between the highest (95% upper bound) TFR estimates and the lowest SRS-based TFR estimates constitutes a 0.446 percent point difference in annual estimated population growth rates. These differences range between a low of 0.21 and a high of 0.73 percentage points (Figure 8). These are clearly not trivial differences and will certainly have an impact upon population dynamic models.

**Table 13:** Annualized Euler-Lotka R by Fertility Estimate.

Fertility Measure	Bihar	Uttar Pradesh	Goa	Madhya Pradesh	Tamil Nadu
SRS	1.06	1.11	0.24	1.34	0.54
Brass PF Original	1.34	1.38	0.99	1.42	1.17
Brass PF Point	1.5	1.54	0.31	1.52	1.21
Brass PF Lower Bound	1.48	1.52	0.12	1.50	1.16
Brass PF Upper Bound	1.52	1.55	0.63	1.55	1.27
Percent Point Difference, SRS vs Upper Bound	0.46	0.44	0.39	0.21	0.73

No gold-standard reference fertility levels exist against which we may benchmark the performance of the stochastic method—or the traditional application for that matter. A number of possibilities exist with respect to the accuracy of the proposed method. First, it is possible that the traditional PF ratio based estimates of age-specific and total fertility are accurate and that the stochastic variants do represent over-estimates of fertility levels. A closer correspondence between the DHS-reported parity of women 45–49 and the traditional Brass-based estimates is supportive of this proposition, but this is likely a mechanistic artifact because it is the DHS data itself which drives the P3/F3 ratio used in the deterministic version of Brass' model. It seems more likely that some aspect of the error propagation associated with the simulation of the distribution of the P3/F3 ratio is responsible for the increased values. Along these lines, inflated values of the P3/F3 ratio could occur if the distributions associated with ASFR estimate are mis-specified. One way this could have occurred in the current analysis is associated with an assumption that the DHS distributions of fertility during the past 12 months would be reflective of the variation associated with the SRS-based estimates—introduced by a lack of available data from the Indian Census on standard deviations of these measurements. In this case, wider than estimated margins of error associated with mis-specified standard deviations could produce the higher than expected stochastic Brass PF ratio-based estimates reported here.

While possible, this shortcoming does not invalidate the thrust of this paper. The focus of here has been illustrative: it provides a clear example with readily-available data of the proposed application. In practical application, any appropriate distribution might be applied to estimating the first-step inputs driving the procedure. Moreover, direct estimates of standard deviations with appropriate sample sizes—rather than surrogate inputs to approximate them—should produce less-inflated estimates of age-specific and total fertility rates. It remains possible that incomplete reporting in the DHS could result in mis-estimation of the surrogate standard deviations utilized in this study—just as easily as the case with the SRS-based estimates. In that case, the inflation could result from the same systematic incompleteness associated with this dataset. Systematic underreporting of fertility can lead to mis-estimation of variance just as easily as the proportion, suggesting that this inflation could as easily be real as artifactual. Without a gold-standard for comparison, this issue will remain unresolved in the current research.

It is clear that the potential inflation of these estimates, however, is not a basic shortcoming related to the use of monte carlo simulation for fitting distributions, which has a long history in statistics, uncertainty analysis, and decision modeling. It is intimately associated with many forms of hypothesis testing in these disciplines [46], [53], [54], [55], [56]. The exercise presented here is largely one of distribution-fitting and monte carlo simulation has been seen as an acceptable alternative to procedures such as jack-knifing or boot-strapping, which involve resampling of an observed distribution of values, when there is strong justification for assuming a particular probability model [13], [43], [53]. These procedures may be used to obtain valid hypothesis tests under these conditions [54], [57]. In the current study, Kolmogorov-Smirnov tests of normality [49], [50] for children-ever-born distributions in each age interval indicated the appropriateness of this distribution for the reported analysis. Given the lack of available data on standard deviations for the age-specific fertility data employed here, no goodness of fit tests could be utilized to verify that the binomial distribution accurately reflects them; however, no known distribution exists with greater conceptual plausibility for capturing the risk of birth than the binomial. Moreover, this distribution has been assumed in a number of other previously-published studies of fertility probability in non-human populations [13], [43], [47], [48]. In spite of the possibility of some inflation of estimates in the current study, there is every indication that the method presented here should produce valid, robust, and accurate estimates of age-specific and total fertility.

The approach presented in this research appears to be a fruitful direction for further development of a stochastic methods for indirect estimation in demography. The paper presents a valid method for estimating the uncertainty associated with Brass PF ratio-based age-specific and total fertility rates. Future evaluations should explore the properties of this method across a larger variety of settings and attempt, where possible, to investigate further whether the method is prone to upward-biasing of these estimates or, in fact, produces more accurate ones. In either case, demographers in need of incorporation of statistical uncertainty into indirect estimation procedures may find the described method here to be a fruitful avenue for application.

## Supporting Information

Figure S1 **Glossary of Terms.**
(DOCX)

Figure S2 **Annotated Sample R Code to Perform the Stochastic Brass PF Ratio Method**
(DOCX)

## References

[1] Shyrock H, Siegel J (1980). The Methods and Materials of Demography. Volume 2.

[2] Keyfitz N, Caswell H (2005). Applied Mathematical Demography. 2^nd^ edition.

[3] Wilson EO, Bossert W (1971). A Primer of Population Biology.

[4] Siegel J, Swanson D (2004). The Methods and Materials of Demography. 2^nd^ edition.

[5] Bryan T, Siegel JacobS, Swanson David (2004). *Population Estimates*.. The Methods and Materials of Demography. 2^nd^ edition.

[6] George MV, Smith SK, Swanson D, Tayman J, Siegel JacobS, Swanson David (2004). *Population Projections*.. The Methods and Materials of Demography. 2^nd^ edition.

[7] Brass W (1964). Uses of Census and Survey Data for the Estimation of Vital Rates..

[8] United Nations (1983). UNM Manual X: Techniques for Indirect Estimation..

[9] Lee D (1969). An Estimation of Level of Fertility in Korea from Special Demographic Survey Data on Births and Children Ever Born.. Yonsei Medical Journal.

[10] Arriaga E, Johnson PD, Jamison E (1994). Population Analysis with Microcomputers..

[11] Coale AJ, Trussell J (1974). Model Fertility Schedules: Variations in the Age Structure of Childbearing in Human Populations.. Population Index.

[12] Wachter K (2006). Essential Demographic Methods.

[13] Caswell H (2001). Matrix Population Models: Construction, Analysis, and Interpretation.

[14] Brass W (1964). Uses of Census and Survey Data for the Estimation of Vital Rates..

[15] Brass W (1965). Methods of Obtaining Basic Demographic Measures where Census and Vital Statistics Registration Systems are Lacking or Defective..

[16] Brass W, Coale AJ, Demeny P, Heisel DF, Lorimer F, Romaniuk A, Van de Walle E (1968). The Demography of Tropical Africa.

[17] Lee D (1969). An Estimation of Level of Fertility in Korea from Special Demographic Survey Data on Births and Children Ever Born.. Yonsei Medical Journal.

[18] Feeney G (1983). Population Dynamics Based on Birth Intervals and Parity Progression.. Population Studies.

[19] Yimamu E (1990). Problems of Selecting a Plausible Fertility Measure for Addis Ababa (Based on the 1984 Census Data).. International Statistical Review.

[20] Mturi A, Hinde A (2001). Fertility Levels and Differentials in Tanzania..

[21] Mitra S (1983). Generalization fo the Immigration the Stable Population Model.. Demography.

[22] Cerone P (1987). On Stable Population Theory with Immigration.. Demography.

[23] Hogan H, Murdock SH, Swanson DA (2008). Measuring Population Change Using the American Community Survey.. Applied Demography in the 21^st^ Century.

[24] Hornberger G, Spear R (1981). An Approach to the Preliminary Analysis of Environmental Systems.. Journal of Environmental Management.

[25] Kulkarni VG (2005). Introduction to the Modeling and Analysis of Stochastic Systems. 2^nd^ Edition.

[26] Sacks J, Welch W, Mitchell T, Wynn H (1989). Design and Analysis of Computer Experiments.. Statistical Science.

[27] Saltelli A, Tarantola S, Campolongo F, Ratto M (2004). Sensitivity Analysis in Practice: A Guide to Assessing Scientific Models.

[28] Saltelli A, Tarantola S, Chan K (1999). Quantitative Model-Independent Method for Global Sensitivity Analysis of Model Output.. Technometrics.

[29] Cox DC, Baybutt P (1981). Methods for Uncertainty Analysis: a Comparative Survey.. Risk Analysis.

[30] Helton J, Johnson J, Sallaberry C, Storlie C (2006). Survey of Sampling-Based Methods for Uncertainty and Sensitivity Analysis.. Reliabilty Engineering & System Safety.

[31] Devroye L (1986). Non-Uniform Random Variate Generation.

[32] Gardiner CW (1983). Handbook of Stochastic Methods for Physics, Chemistry, and the Natural Sciences.

[33] Kalos MH, Whitlock PA (1986). Monte Carlo Methods: Basics.

[34] Doll JD, Freeman DL (1986). Randomly Exact Methods.. Science.

[35] Fishman GS (1986). Monte Carlo: Concepts, Algorithms, and Applications.

[36] Rubinstein RY (1981). Simulation and the Monte Carlo Method.

[37] Sobol IM (1994). A Primer for the Monte Carlo Method.

[38] Dong WM, Chiang WL, Wong FS (1987). Propagation of Uncertainties in Deterministic Systems.. Computers and Structures.

[39] Goodman D (1984). Statistics of Reproductive Rate Estimates and Their Implications for Population Projection.. Reports of the International Whaling Commission, Special Issue.

[40] Barnthouse W, Suter G, Rosen A (1990). Risks of Toxic Contaminants to Exploited Fish Populations: Influence of Life-History, Data Uncertainty, and Exploitation Intensity.. Environmental Toxicology and Chemistry.

[41] Ragen T (1995). Maximum Net Productivity Level Estimation for the Northern Fur Seal (*Callorhinus ursinus*) Population of St. Paul Island Alaska.. Marine Mammal Science.

[42] Powell RA, Zimmerman J, Seaman D, Gilliam J (1996). Demographic Analysis of a Hunted Black Bear Population With Access to Refuge.. Conservation Biology.

[43] Caswell H, Brault S, Read A, Smith T (1998). Harbor Porpoise and Fisheries: An Uncertainty Analysis of Incidental Mortality.. Ecological Applications.

[44] Lee R, Tuljapurkar S (1994). Stochastic Population Forecasts of the US: Beyond High, Medium, and Low.. Journal of the American Statistical Association.

[45] Brown LD, Cai T, DasGupta A (2001). Interval Estimation for a Binomial Proportion.. Statistical Science.

[46] Smith J (1993). Moment Methods for Decision Analysis.. Management Science.

[47] Jonzen N, Pople T, Knape J, Skold M (2010). Stochastic Demography and Population Dynamics in the Red Kangaroo *Macropus rufus*.. Journal of Animal Ecology.

[48] Linstrom J, Reeve R, Salvidio S (2010). Bayesian Salamanders: Analyzing the Demography of an Underground Population of the European Plethodontid *Speleomante sstrinatii* With State-Space Modeling.. BMC Ecology.

[49] Samuels M, Witmer J (1998). Statistics for the Life Sciences.

[50] Massey FT (1951). The Kolmogorov-Smirnov Test for Goodness of Fit.. Journal of the American Statistical Association.

[51] Little R, Rubin D (1987). Statistical Analysis with Missing Data. 2^nd^ Edition.

[52] Schafer J (1999). Multiple Imputation: A primer.. Statistical Methods in Medical Research.

[53] Dwass M (1957). Modified Randomization Tests for Nonparametric Hypotheses.. Annals of Mathematical Statistics.

[54] Barnard GA (1963). Discussion of Professor Bartlett's Paper. Journal of the Royal Statistical Society.. Series B (Methodological).

[55] Jockel K (1986). Finite Sample Properties and Asymptotic Efficiency of Monte Carlo Tests.. The Annals of Statistics.

[56] Besag J, Clifford P (1989). Generalized Monte Carlo Significance Tests.. Biometrika.

[57] Ramberg J, Dudewicz E, Tadikamalla P, Mykytka E (1979). A Probability Distribution and its Uses to Fitting Data.. Technometrics.

