# Supplementary material for: A Stochastic Version of the Brass PF Ratio Adjustment of Age-Specific Fertility Schedules
Source: PLoS One. 2011 Aug 4;6(8):e23222. doi: 10.1371/journal.pone.0023222 (PMC3150419; doi:10.1371/journal.pone.0023222)
Supplement: Figure S1 — Glossary of Terms. (DOCX) [file pone.0023222.s001.docx]

**S1. Glossary of Terms**

Age-specific Fertility Rate (ASFR): A measure of the risk of experiencing a live birth within a given age-interval. Mathematically, the ASFR is the number of births within an age-interval, divided by number of women at risk for giving birth.

Children Ever Born. Measure of *parity*, the number of live births experienced at a given age. In this paper, expressed as a normally-distibuted variable characterized by a specific mean and variance, within each age-group from 15-19 to 45-49 years.

Cumulative Fertility. The partial sum of age-specific fertility rates, up to the age-group of interest. For example, the cumulative fertility at age 35-39 would be the sum of age-specific fertilities for the intervals 15-19, 20-24, 25-29, 30-34, and 35-39.

Parity. The number of live births to a given woman of a specific age.

Total Fertility Rate. Technically, the sum of age-specific fertility rates from the youngest interval to the oldest—typically 15-19 to 45-49.
