# Supplementary material for: A Stochastic Version of the Brass PF Ratio Adjustment of Age-Specific Fertility Schedules
Source: PLoS One. 2011 Aug 4;6(8):e23222. doi: 10.1371/journal.pone.0023222 (PMC3150419; doi:10.1371/journal.pone.0023222)
Supplement: Figure S2 — Annotated Sample R Code to Perform the Stochastic Brass PF Ratio Method (DOCX) [file pone.0023222.s002.docx]

**S2. An Annotated Sample R Code for the Stochastic Brass PF Ratio**

**##This reads in the survey-based estimates of age-specific fertility as a .csv file##**

ASFR<-read.table(file="...*provide path here*.csv",sep=",")

**##This produces 10K realizations of the survey-based estimates of the distribution of age-specific fertiity by age, using the normal approximation of the binomial distribution##**

ASFR1519sim<-rnorm(# monte carlo samples, proportion, standard deviation)

ASFR2024sim<-rnorm(# monte carlo samples, proportion, standard deviation)

.

.

.

ASFR4549sim<-rnorm# monte carlo samples, proportion, standard deviation)

**##This produces 10K realizations of partial sums of cumulative fertility up to each 25-29 age interval, assuming a normal distribution##**

CumF2529<-sum(ASFR1519,ASFR2024,ASFR2529)

**##This produces 10K realizations of Parity for each age group##**

CEB1519<-rnorm(# monte carlo samples, mean, standard deviation)

CEB2024<-rnorm(# monte carlo samples, mean, standard deviation)

CEB2529<-rnorm(# monte carlo samples, mean, standard deviation)

.

.

.

CEB4549<-rnorm(# monte carlo samples, mean, standard deviation)

**##This produces 10K realizations of the P3/F3 ratio##**

P3F3sim<-CEB2529/CumF2529

**##This adjusts the ASFR schedule according to the average P3/F3 ratio##**

P3F3<-mean(P3F3)

AdjASFR<-ASFR*P3F3

**##This writes the final adjusted estimates out to a .csv file##**

PFASFR<-write.table(file=". . .*provide path here*.csv",sep=",")
